# Prozone masks elevated SARS-CoV-2 antibody level measurements

**DOI:** 10.1371/journal.pone.0301232

**Published:** 2024-03-28

**Authors:** Micaela N. Sandoval, Samuel P. McClellan, Stephen J. Pont, Jessica A. Ross, Michael D. Swartz, Mark A. Silberman, Eric Boerwinkle

**Affiliations:** 1 Department of Epidemiology, Human Genetics, and Environmental Sciences, The University of Texas Health Science Center at Houston School of Public Health, Houston, Texas, United States of America; 2 Clinical Pathology Laboratories, Austin, Texas, United States of America; 3 Center for Public Health Policy and Practice at Texas Department of State Health Services, Austin, Texas, United States of America; 4 Department of Biostatistics and Data Science, The University of Texas Health Science Center at Houston School of Public Health, Houston, Texas, United States of America; Makerere University Medical School and the National Malaria Control Division, UGANDA

## Abstract

We report a prozone effect in measurement of SARS-CoV-2 spike protein antibody levels from an antibody surveillance program. Briefly, the prozone effect occurs in immunoassays when excessively high antibody concentration disrupts the immune complex formation, resulting in a spuriously low reported result. Following participant inquiries, we observed anomalously low measurement of SARS-CoV-2 spike protein antibody levels using the Roche Elecsys® Anti-SARS-CoV-2 S immunoassay from participants in the Texas Coronavirus Antibody Research survey (Texas CARES), an ongoing prospective, longitudinal antibody surveillance program. In July, 2022, samples were collected from ten participants with anomalously low results for serial dilution studies, and a prozone effect was confirmed. From October, 2022 to March, 2023, serial dilution of samples detected 74 additional cases of prozone out of 1,720 participants’ samples. Prozone effect may affect clinical management of at-risk populations repeatedly exposed to SARS-CoV-2 spike protein through multiple immunizations or serial infections, making awareness and mitigation of this issue paramount.

## Introduction

Measures of SARS-CoV-2 antibody levels, such as those reported in multiple publications [1–3], are used to determine whether or not a person has been exposed to an antigen, characterize the extent of an individual’s immune response, and to infer rates of both infection and vaccination in populations [4–6]. The prozone effect occurs in immunoassays when excessively high antibody concentration oversaturates and disrupts the antigen-antibody bridging between immobile phase and chemiluminescent signal, resulting in a spuriously low reported result. This phenomenon is well documented in serological testing of other infectious diseases, including syphilis, HIV, and malaria [7,8]. Here, we report a prozone or ‘hook’ effect adversely influencing measurement of SARS-CoV-2 spike protein antibody levels using the Roche Elecsys® Anti-SARS-CoV-2 S immunoassay from participants in the Texas Coronavirus Antibody Research survey (Texas CARES), an ongoing prospective, longitudinal antibody surveillance program.

## Materials and methods

### Study population

The Texas CARES population and study design are described in detail elsewhere [9,10]; the program began enrolling a convenience sample of adults and children from across Texas in October 2020. Participants provided serum samples at approximately three-month intervals, which were tested for anti-SARS-CoV-2 spike and nucleocapsid proteins using the Roche Elecsys® Anti-SARS-CoV-2 quantitative S test and qualitative N test assays, respectively. Participants completed questionnaires at each test timepoint to capture demographic and clinical characteristics, including COVID-19 infection history, vaccination status, and medical history. The program includes innovative return of results processes to inform and engage study participants. As of February 2023, Texas CARES has enrolled 90,011 participants, of whom 40,572 have completed at least four antibody testing timepoints.

### Initial discovery of anomalous anti-SARS-CoV-2 spike protein antibody results

Beginning in late November of 2021, certain participants contacted program staff with concerns about their Anti-SARS-CoV-2 spike protein results, having noticed a precipitous decrease in reported antibody concentration between consecutive test dates, inconsistent with expected antibody decay. In response to participant concerns, the program conducted a quality audit, in which 40 participants’ samples collected from November 2021 –February 2022, were retested according to the original Roche instructions for use (IFU) [11]. All retested samples produced antibody levels consistent with the original result, excluding laboratory error as the source of the anomalous results. Investigators considered and excluded mitigating factors from natural or artificial immunosuppression through dialogues with affected participants. The program found that affected participants frequently reported natural infections and/or immunization boosters and had previously reported high antibody results, triggering the consideration of a (at the time) previously uncharacterized prozone effect.

### Exploratory dilution studies

To evaluate possible hook effect in 3 specimens that reproducibly tested in the analytical measurable range (AMR) of the assay (0.4–250 U/mL with onboard 10X automated dilution, clinical reportable range (CRR): 0.4–2500 U/mL), the laboratory re-evaluated with initial predilution of 1:5 and 1:50 prior to testing in accordance with the IFU. Final endpoint results of samples pre-diluted at 1:50 were above the clinical reportable maximum of 2500 U/mL, supporting the prozone effect theory. In August 2022, the program conducted an additional confirmation study, identifying participants who had recently received low antibody titers (0.8–250 U/mL reported result) immediately following a high antibody titer (>2500 U/mL reported result). Study staff then requested new serum samples from eleven of these participants for repeat testing using the 1:50 pre-dilution protocol.

### Implementation of standardized serial dilution protocol

**Fig 1:**
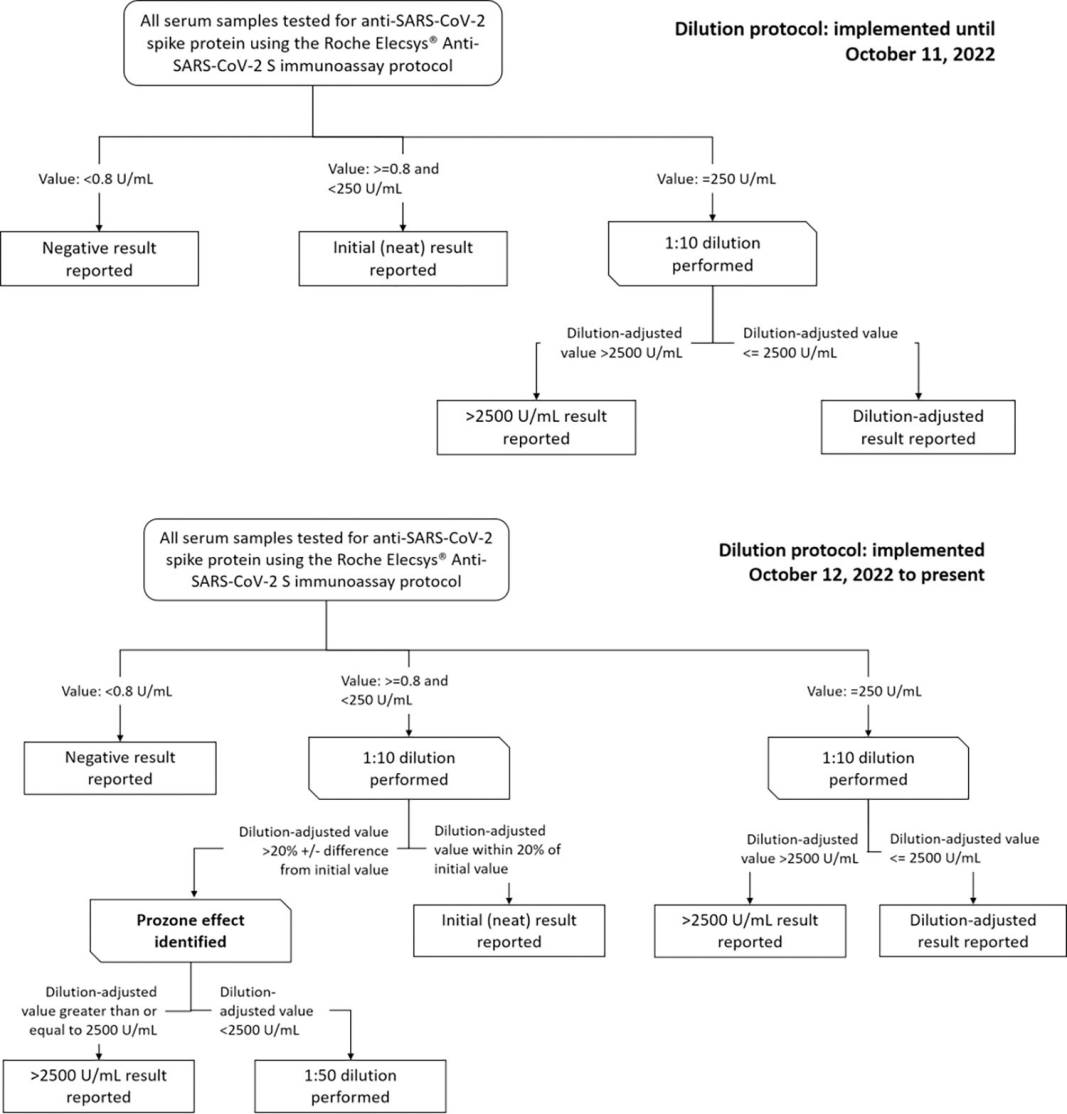
Dilution protocol for anti-SARS-CoV-2 spike protein immunoassay, implemented October 12, 2022.

Beginning October 12, 2022, all study samples with initial neat results of 0.8–250 U/mL were subjected to a dilution algorithm with initial retest at 1:10 and reviewed for possible prozone effect (Fig 1). Initial (neat) results greater than or equal to 0.8 U/mL and less than 250 U/mL underwent 1:10 dilution. The dilution-adjusted values were compared to the initial neat values, and if the two values were within 20% of each other, the initial neat result was reported. If the dilution-adjusted value exhibited a greater than 20% difference from the initial neat value, the sample was classified as a prozone case. Among these prozone cases, if the dilution-adjusted value was greater than or equal to 2500 U/mL, a value of >2500 U/mL was reported to participants; if the dilution-adjusted value was less than 2500 U/mL, an additional 1:50 dilution was performed to rule out persistent prozone effect.

### Statistical analyses

Laboratory values were reported as the median and interquartile range (IQR) for continuous variables. All analyses were performed on Stata MP version 17.0 (StataCorp LLC, College Station, TX, USA).

### Ethics statement

All protocols were reviewed and approved by the UTHealth Committee for Protection of Human Subjects, Institutional Review Board: # HSC-SPH-20-0825, but also were deemed public health practice by the Texas Department of State Health Services IRB. All adults consented electronically to be in the study, and children under 18 either consented or assented to be in the study as appropriate for their age. The catchment area for the cohort study was the entire state of Texas. Recruitment efforts were taken to enroll rural and urban participants spanning the state. More details about the study are publicly available on the Texas CARES dashboard [12].

## Results and discussion

The results of the exploratory dilution studies are presented in Table 1. Participants A, B, and C represent the three samples first retested under the 1:50 pre-dilution procedure: final endpoint results of these ranged from 8,600 to 66,100 U/mL. Of the eleven participants asked to submit additional serum samples, seven provided new samples. These seven participants’ (D-J in Table 1) samples demonstrated prozone effect using 1:50 predilution with final antibody levels ranging from 3,845 to 20,450 U/mL as given in Table 1.

**Table 1:** Prozone effect in anti-SARS-CoV-2 spike protein immunoassay testing, August 2022.

ID	Age	Sex	Vaccination status		COVID-19 infection history		Initial (neat) result	1:50 predilution result
	(years)		Participant-reported immunization history	Time from latest immunization to test (days)	Participant-reported infection history	Time from latest infection to test (days)	(U/mL)	(U/mL)
A*	60–69	Male	2x full dose, 2x booster	36	None		201	8,600
B*	60–69	Female	2x full dose, 2x booster	19	None		220	11,600
C*	70–79	Female	2x full dose, 2x booster	30	None		144	66,100
D	50–59	Male	2x full dose, 2x booster	145	None		181	3,845
E	60–69	Female	2x full dose, 2x booster	124	1x infection	242	240	20,450
F	70–79	Male	2x full dose, 2x booster	65	None		211	8,100
G	70–79	Female	2x full dose, 2x booster	103	None		196	>12,500†
H	70–79	Male	2x full dose, 2x booster	149	None		198	>12,500†
I	70–79	Female	3x full dose, 1x booster	184	1x infection	27	205	>12,500†
J	50–59	Female	2x full dose, 2x booster	71	None		211	>12,500†

* Samples from participants A, B, and C were retained and retested at 1:5 and 1:50 predilution prior to testing according to instructions for use.

†As protocol was developed, laboratory finalized antibody quantitation at >12,500 U/mL rather than diluting to endpoint concentration.

Note: all corrections of prior results and instances of newly identified hook were reported to participants as >2,500 U/mL to align with program historical resulting.

Following implementation of the standardized serial dilution protocol in October 2022, additional prozone cases were identified. Between October 12, 2022, and February 28, 2023, there were 74 additional instances of prozone effect detected out of 1,720 participants’ samples. Median initial (neat) result for these 74 samples was 177 U/mL, interquartile range: 122 U/mL to 210 U/mL (S1 Table). Following 1:10 predilution and retesting, all 74 samples had final (reported) results of >2,500 U/mL, in accordance with the updated reporting protocol.

## Discussion

The results of this report add to the growing body of evidence suggesting that the prozone effect may artificially decrease anti-SARS-CoV-2 spike antibody titer results in certain immunoassays [13–15]. To the best of our knowledge, the prozone effect resulted in spuriously low positive results but did not produce a negative antibody result using the Roche Elecsys S antibody assay in the Texas CARES program. The consistency of our findings with those of independent contemporaneous investigations such as those published by Abedon, et al. [13] are indicative of the scale and clinical relevance of this phenomenon. As COVID-19 becomes endemic globally and immunization protocols evolve, people experiencing numerous successive natural and/or artificial SARS-CoV-2 exposures, may undergo more extreme immune responses. In response to the rapid increase in reported levels of anti-SARS-CoV-2 antibody titers, some laboratory protocols now report results up to 250,000 U/mL [16]. While a clinical antibody-based threshold of protection against COVID-19 infection has not yet been defined, serological results remain important in clinical decision-making, such as vaccination protocols for immunosuppressed patients.

## Conclusion

The probability of a prozone effect obscuring high antibody concentrations will certainly only increase as populations become repeatedly exposed to SARS-CoV-2 spike protein through multiple immunizations or serial infections, making awareness and mitigation of this issue paramount. To address the likelihood of prozone effect obscuring very high antibody titer results, laboratories should be aware of this phenomenon to consider implementing defined and standardized dilution protocols as appropriate for clinical, research, and public health applications.

## Supporting information

S1 Table Prozone cases identified within Texas CARES study, October 12, 2022 to February 28, 2023. (DOCX)
