# Supplementary material for: Prozone masks elevated SARS-CoV-2 antibody level measurements
Source: PLoS One. 2024 Mar 28;19(3):e0301232. doi: 10.1371/journal.pone.0301232 (PMC10977713; doi:10.1371/journal.pone.0301232)
Supplement: S1 Table — (DOCX) [file pone.0301232.s001.docx]

Supplement 1.

| Supplementary Table 1. Prozone cases identified within Texas CARES study, October 12, 2022 to February 28, 2023 | | | |
| --- | --- | --- | --- |
| De-identified participant number | Initial (neat) result (U/mL) | Pre-dilution factor | Final (reported) result (U/mL) |
| 1 | 231 | 1:10 | >2500 |
| 2 | 170 | 1:10 | >2500 |
| 3 | 248 | 1:10 | >2500 |
| 4 | 246 | 1:10 | >2500 |
| 5 | 173 | 1:10 | >2500 |
| 6 | 99.5 | 1:10 | >2500 |
| 7 | 158 | 1:10 | >2500 |
| 8 | 131 | 1:10 | >2500 |
| 9 | 133 | 1:10 | >2500 |
| 10 | 210 | 1:10 | >2500 |
| 11 | 249 | 1:10 | >2500 |
| 12 | 201 | 1:10 | >2500 |
| 13 | 206 | 1:10 | >2500 |
| 14 | 212 | 1:10 | >2500 |
| 15 | 223 | 1:10 | >2500 |
| 16 | 188 | 1:10 | >2500 |
| 17 | 200 | 1:10 | >2500 |
| 18 | 125 | 1:10 | >2500 |
| 19 | 77.7 | 1:10 | >2500 |
| 20 | 95.2 | 1:10 | >2500 |
| 21 | 176 | 1:10 | >2500 |
| 22 | 162 | 1:10 | >2500 |
| 23 | 162 | 1:10 | >2500 |
| 24 | 241 | 1:10 | >2500 |
| 25 | 230 | 1:10 | >2500 |
| 26 | 118 | 1:10 | >2500 |
| 27 | 164 | 1:10 | >2500 |
| 28 | 122 | 1:10 | >2500 |
| 29 | 229 | 1:10 | >2500 |
| 30 | 193 | 1:10 | >2500 |
| 31 | 164 | 1:10 | >2500 |
| 32 | 180 | 1:10 | >2500 |
| 33 | 75.1 | 1:10 | >2500 |
| 34 | 99.3 | 1:10 | >2500 |
| 35 | 91.7 | 1:10 | >2500 |
| 36 | 194 | 1:10 | >2500 |
| 37 | 191 | 1:10 | >2500 |
| 38 | 197 | 1:10 | >2500 |
| 39 | 97.3 | 1:10 | >2500 |
| 40 | 181 | 1:10 | >2500 |
| 41 | 152 | 1:10 | >2500 |
| 42 | 211 | 1:10 | >2500 |
| 43 | 109 | 1:10 | >2500 |
| 44 | 113 | 1:10 | >2500 |
| 45 | 247 | 1:10 | >2500 |
| 46 | 136 | 1:10 | >2500 |
| 47 | 60.1 | 1:10 | >2500 |
| 48 | 119 | 1:10 | >2500 |
| 49 | 69 | 1:10 | >2500 |
| 50 | 248 | 1:10 | >2500 |
| 51 | 228 | 1:10 | >2500 |
| 52 | 102 | 1:10 | >2500 |
| 53 | 100 | 1:10 | >2500 |
| 54 | 177 | 1:10 | >2500 |
| 55 | 179 | 1:10 | >2500 |
| 56 | 155 | 1:10 | >2500 |
| 57 | 164 | 1:10 | >2500 |
| 58 | 242 | 1:10 | >2500 |
| 59 | 203 | 1:10 | >2500 |
| 60 | 172 | 1:10 | >2500 |
| 61 | 180 | 1:10 | >2500 |
| 62 | 215 | 1:10 | >2500 |
| 63 | 197 | 1:10 | >2500 |
| 64 | 194 | 1:10 | >2500 |
| 65 | 89.7 | 1:10 | >2500 |
| 66 | 215 | 1:10 | >2500 |
| 67 | 117 | 1:10 | >2500 |
| 68 | 214 | 1:10 | >2500 |
| 69 | 177 | 1:10 | >2500 |
| 70 | 112 | 1:10 | >2500 |
| 71 | 204 | 1:10 | >2500 |
| 72 | 237 | 1:10 | >2500 |
| 73 | 147 | 1:10 | >2500 |
| 74 | 207 | 1:10 | >2500 |
